# Investigation of interactions between TLR2, MyD88 and TIRAP by bioluminescence resonance energy transfer is hampered by artefacts of protein overexpression

**DOI:** 10.1371/journal.pone.0202408

**Published:** 2018-08-23

**Authors:** Natália G. Sampaio, Martina Kocan, Louis Schofield, Kevin D. G. Pfleger, Emily M. Eriksson

**Affiliations:** 1 Population Health and Immunity Division, Walter and Eliza Hall Institute of Medical Research, Parkville, Victoria, Australia; 2 Department of Medical Biology, University of Melbourne, Parkville, Victoria, Australia; 3 Drug Discovery Biology, Monash Institute of Pharmaceutical Sciences, Monash University, Parkville, Victoria, Australia; 4 Australian Institute of Tropical Health and Medicine, James Cook University, Townsville, Queensland, Australia; 5 Harry Perkins Institute of Medical Research and Centre for Medical Research, The University of Western Australia, Nedlands, Western Australia, Australia; 6 Dimerix Limited, Nedlands, Western Australia, Australia; Virginia Polytechnic Institute and State University, UNITED STATES

## Abstract

Toll like receptors (TLRs) are important pattern recognition receptors that can detect pathogen and danger associated molecular patterns to initiate an innate immune response. TLR1 and 2 heterodimerize at the plasma membrane upon binding to triacylated lipopeptides from bacterial cell walls, or to the synthetic ligand Pam3CSK4. TLR1/2 dimers interact with adaptor molecules TIRAP and MyD88 to initiate a signalling cascade that leads to activation of key transcription factors, including NF-kB. Despite TLRs being extensively studied over the last two decades, the real-time kinetics of ligand binding and receptor activation remains largely unexplored. We aimed to study the kinetics of TLR activation and recruitment of adaptors, using TLR1/2 dimer interactions with adaptors MyD88 and TIRAP. Bioluminescence resonance energy transfer (BRET) allows detection of real-time protein-protein interactions in living cells, and was applied to study adaptor recruitment to TLRs. Energy transfer showed interactions between TLR2 and TIRAP, and between TLR2 and MyD88 only in the presence of TIRAP. Quantitative BRET and confocal microscopy confirmed that TIRAP is necessary for MyD88 interaction with TLR2. Furthermore, constitutive proximity between the proteins in the absence of Pam3CSK4 stimulation was observed with BRET, and was not abrogated with lowered protein expression, changes in protein tagging strategies, or use of the brighter NanoLuc luciferase. However, co-immunoprecipitation studies did not demonstrate constitutive interaction between these proteins, suggesting that the interaction observed with BRET likely represents artefacts of protein overexpression. Thus, caution should be taken when utilizing protein overexpression in BRET studies and in investigations of the TLR pathway.

## Introduction

The main mechanism by which innate immune cells detect foreign pathogens is through pattern recognition receptors. These receptors recognise danger- or pathogen-associated molecular patterns (DAMPs or PAMPs), which are normally absent in the healthy host. Recognition initiates a signalling cascade that stimulates the cell to respond through both inflammatory cytokine release and cellular activation to mediate pathogen destruction. Toll-like receptors (TLRs) are a well characterized group of pattern recognition receptors that play a crucial role in the initial detection of pathogens by the innate immune system [1, 2]. TLRs are transmembrane glycoproteins with a ligand-binding domain in the extracellular N-terminus, and a downstream signalling domain in the intracellular C-terminus. These receptors are proposed to dimerize upon ligand binding, wherein the C-terminal regions of the receptors are brought into contact, and activate signalling through interaction with adaptor proteins [3, 4]. The signal transduction from TLRs to their binding partners occurs via the Toll-interleukin-1 receptor (TIR) domain, which is present in both TLRs and adaptors [2]. TLRs activate downstream signalling cascades that lead to activation of transcription factors, such as nuclear factor kappa light-chain enhancer of activated B cells (NF-κB), activator protein-1 (AP-1), and interferon regulatory factors (IRFs), which in turn initiate a pro-inflammatory response [4].

Humans encode ten TLRs, which detect different PAMPs and DAMPs. TLR2 heterodimerizes with TLR1 or TLR6 to detect triacyl or diacyl lipopeptides, respectively [3, 5]. These receptors are essential for immune responses to Gram-positive and Gram-negative bacteria, and are also involved in detection of PAMPs from fungi and parasites like *Plasmodium* and *Trypanosoma cruzi*. All TLRs, with the exception of TLR3, signal through adaptor Myeloid differentiation factor 88 (MyD88). Additionally, both MyD88 and TIR domain containing adaptor protein (TIRAP, MAL) are necessary for signal transduction from TLR2 and TLR4 [4, 6]. TIRAP directly interacts with MyD88 [7, 8], and contains a phosphatidylinositol 4,5-bisphosphate (PIP_2_) binding site that localizes it to the plasma membrane [9]. TIRAP therefore serves as a bridging adaptor between MyD88 and TLR2, recruiting MyD88 to the plasma membrane where it can be activated by concomitant TLRs. Upon receptor activation, MyD88 is recruited and subsequently interacts with members of the IL-1R-associated kinase (IRAK) family via its death domain [10, 11], to form a multimeric signalling platform called the Myddosome [12]. This in turn triggers downstream signalling that results in NF-κB activation [6].

Due to their central role in pathogen detection, TLRs are targets of pathogen inhibition in order to avoid detection. There are numerous examples of viral and bacterial proteins that inhibit TLR signalling [13–16]. These can target in particular the interaction between TLRs and adaptor molecules. Furthermore, overstimulation of immune responses can also be detrimental to the host. DAMPs released by damaged cells can stimulate TLRs and initiate a positive feedback loop to cause excessive inflammation. TLRs are increasingly being associated with inflammatory diseases such as rheumatoid arthritis, systemic lupus erythematosus, and atherosclerosis [17, 18]. Thus, the development of therapies that specifically target the interaction between TLRs and adaptors to modulate signalling could have important therapeutic applications. However, previous studies of TLR-adaptor interactions and of pathogen TLR-modulating proteins have investigated activation of more general downstream pathways, such as NF-κB activation, mitogen activated protein kinase (MAPK) activity, and cytokine secretion [6, 19, 20]. Since several extracellular stimuli and/or surface receptors converge on these downstream pathways, it is difficult to dissect the exact interaction that is causing the measured effect. A more specific and sensitive method to monitor the interactions between TLRs and adaptors could provide a better understanding of potency of ligand binding, and allow the investigation of molecules that specifically affect the interactions between TLRs and adaptor molecules.

Bioluminescence resonance energy transfer (BRET) is a technique for real-time monitoring of protein-protein interactions in live cells. It has been extensively used to study G protein-coupled receptors [21–23], but to our knowledge has never been adapted for use with TLRs. In BRET, proteins of interest are tagged with either a luciferase enzyme or a fluorophore, and expressed in target cells. Upon treatment with the luciferase substrate, light is emitted from the luciferase at a specific wavelength. If the ‘donor’ luciferase-tagged protein comes within close proximity to the ‘acceptor’ fluorophore-tagged protein, less light is emitted from the luciferase and resonance energy transfer excites the fluorophore, resulting in light emission from the fluorophore at a different wavelength. Energy transfer is indicative of direct interactions between the tagged proteins, as it only occurs when the proteins are within about 8 nm of each other [24]. Therefore, BRET has the potential to allow specific and sensitive detection of the interactions between TLRs and adaptor proteins. We aimed to apply BRET to study the kinetics of TLR and adaptor interaction in live cells, using TLR1/2 binding to TIRAP and MyD88 as a model system.

## Results

### Tagged TLR1/2 and TIRAP, but not MyD88, localize to the plasma membrane

**Fig 1:**
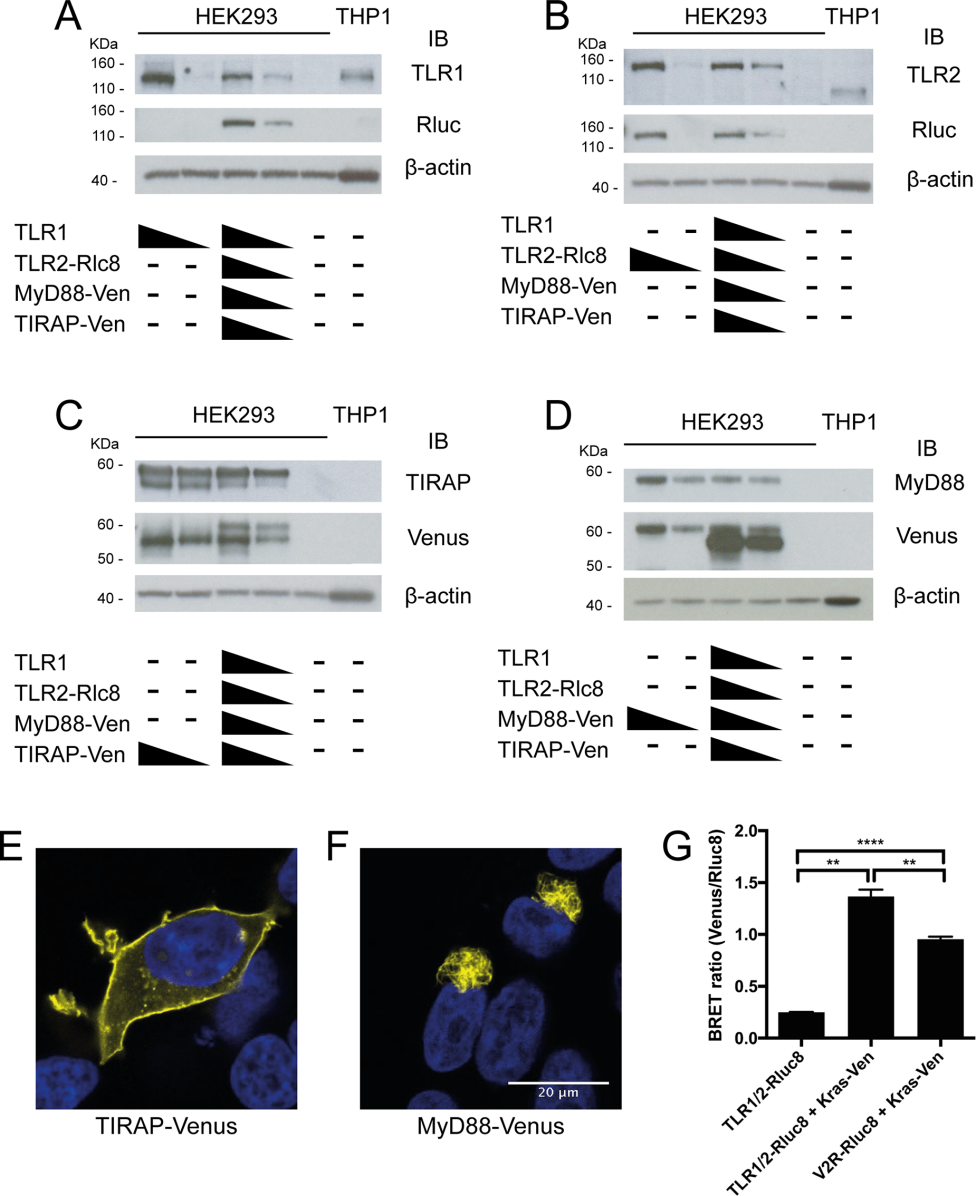
BRET constructs are functional and appear to be appropriately localized when transfected into cells. A-D) HEK293FT cells were transfected with 0, 30 or 300 ng of TLR1, TLR2-Rluc8, TIRAP-Venus or MyD88-Venus constructs, alone or in combination, and analysed for protein expression by Western immunoblot (IB) as indicated, along with lysate from untransfected THP1 cells. HEK293FT cells were transfected with E) TIRAP-Venus or F) MyD88-Venus constructs, DAPI stained, and imaged by confocal microscopy. Yellow = Venus, blue = nucleus; representative images of two independent experiments. G) HEK293FT cells were transfected with 100 ng TLR1/2-Rluc8 construct only (negative control), 100 ng TLR1/2-Rluc8 + 100 ng Kras-Venus constructs, and 100 ng V2R-Rluc8 + 100 ng Kras-Venus constructs (positive control) and BRET ratio was measured. Error bars represent SD of three independent experiments. One-way ANOVA; **** = *P*<0.0001, ** = *P*<0.01.

In order to investigate the real-time kinetics of TLR1/2 activation in live cells by BRET, TLR1 was left untagged, TLR2 was tagged with donor luciferase (Rluc8), and TIRAP or MyD88 was tagged with the acceptor fluorophore (Venus). This combination of luciferase and fluorophore has been previously demonstrated to perform well in BRET experiments [25]. Western blot analysis of HEK293FT cells transfected with these DNA expression constructs showed that the tagged proteins were co-expressed in a dose-dependent manner and at the expected molecular weight (Fig 1A–1D). Confocal microscopy of cells transfected with MyD88-Venus or TIRAP-Venus constructs showed that TIRAP-Venus localized mostly to the plasma membrane, as was expected (Fig 1E), whereas MyD88 was found in condensed intracellular structures (Fig 1F), consistent with that previously reported for overexpressed MyD88 [9, 26–28].

In order to determine if TLR2 was localized to the plasma membrane upon expression in cells, TLR1 and TLR2-Rluc8 constructs were co-transfected with Kras-Venus construct and BRET was measured. Kras-Venus contains only the C-terminal fragment of Kras N-terminally linked to Venus, and when overexpressed in cells, it coats the plasma membrane with Venus fluorophore to function as a plasma membrane ‘tag’ for BRET [29]. The BRET ratio is determined by dividing light emission at wavelengths characteristic of the fluorophore’s emission peak by light emission characteristic of the luciferase’s emission peak. When TLR1/2-Rluc8 was co-transfected with Kras-Venus, a significant increase in BRET ratio was observed (1.33) compared to TLR1/2-Rluc8 only (0.25), indicating the receptors were plasma membrane-localized (Fig 1G). Indeed, the BRET ratio was significantly greater than that observed with Vasopressin-2-receptor (V2R)-Rluc8, a plasma membrane-localized GPCR previously assessed using BRET [22] that was used as a positive control. Thus, tagged TLR2, MyD88, and TIRAP were successfully ectopically expressed in HEK293FT cells, and displayed the expected subcellular localization.

### BRET shows ligand-independent interaction between TLR1/2 and adaptors

**Fig 2:**
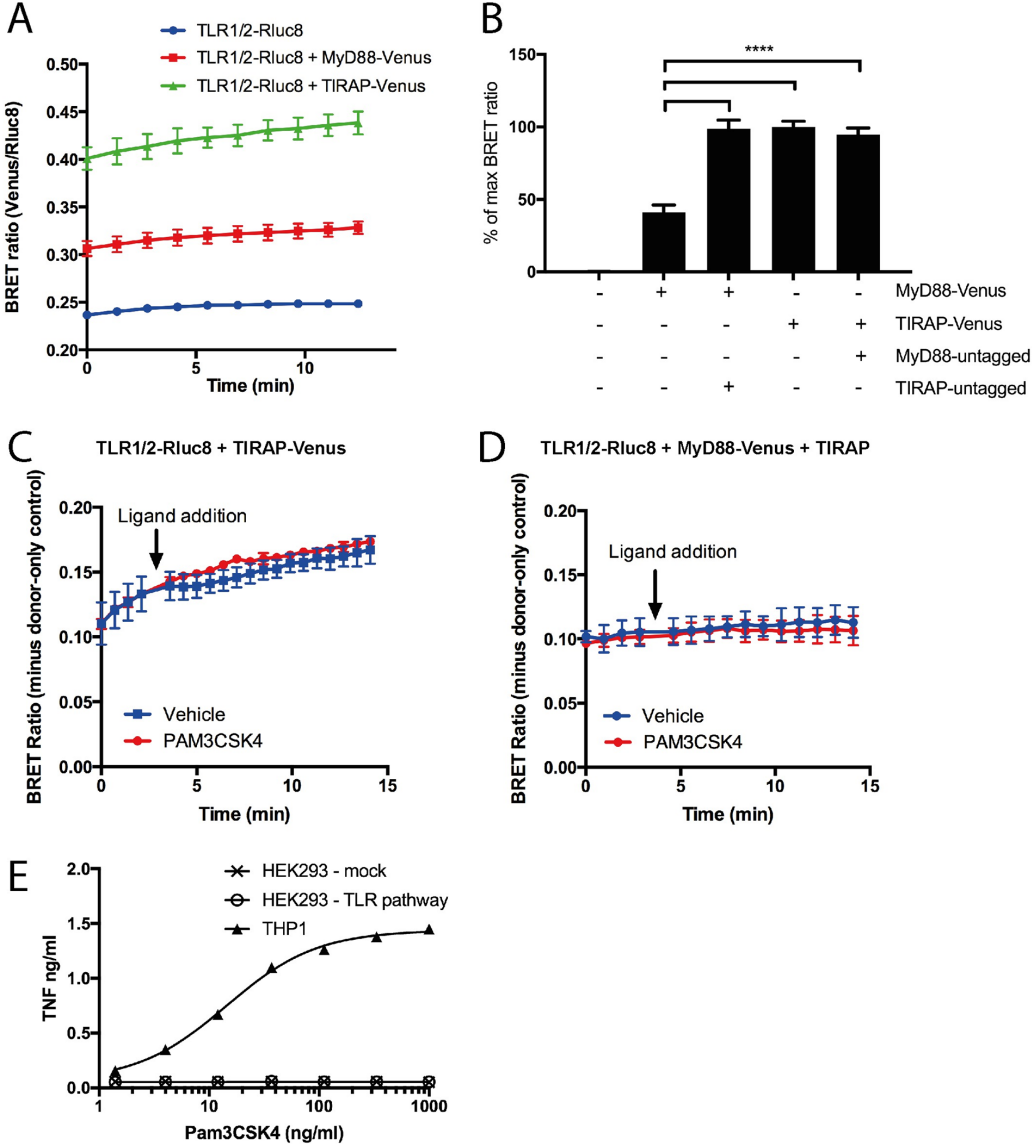
TIRAP directly interacts with TLR1/2, but does not show ligand-induced BRET increase. A) HEK293FT cells were transfected with 100 ng TLR1 and 100 ng TLR2-Rluc8 constructs, and additionally with 300 ng MyD88-Venus, or 300 ng TIRAP-Venus constructs, and BRET measured over time. Graph shown is representative of three independent experiments; error bars represent SD of triplicate wells. B) Transfections as in (A), with additional cells transfected with 300 ng MyD88-Venus + 300 ng TIRAP constructs, or 300 ng MyD88 + 300 ng TIRAP-Venus constructs. Data shown as % of max BRET, where max is TLR1/2-Rluc8 + TIRAP-Venus; n = 4 independent experiments. One-way ANOVA; **** = *P*<0.0001. HEK293FT cells transfected with 300 ng TLR1 + 300 ng TLR2-Rluc8 constructs and additionally with either C) 300 ng TIRAP-Venus or D) 100 ng MyD88-Venus + 300 ng of TIRAP constructs. Cells were stimulated with 10 μg/ml Pam3CSK4 or vehicle control and BRET measured over time. Data shown are donor-only-subtracted BRET ratio; graphs are representative of three independent experiments. E) Untransfected THP1 cells, or HEK293FT cells transfected with 0 or 300 ng of TLR1/2, MyD88 and TIRAP constructs (TLR pathway), were treated with increasing concentrations of Pam3CSK4, as indicated, and TNF was measured in culture medium after 24 hr. Dose-response curve was fitted using Graphpad Prism non-linear fit with variable slope (EC50 = 14.4 for THP1 cells).

TLR1/2-Rluc8 co-expression with TIRAP-Venus or MyD88-Venus caused an increase in BRET ratio, when compared to TLR1/2-Rluc8 alone (Fig 2A). Note that background BRET ratio is caused by luminescence detection in the ‘fluorescence’ channel, and has been subtracted from all subsequent BRET graphs. When BRET ratios with MyD88-Venus and TIRAP-Venus were compared (Fig 2B), MyD88-Venus was found to result in a significantly lower BRET ratio than TIRAP-Venus. However, if untagged TIRAP was co-expressed with MyD88-Venus, BRET increased to levels equivalent to that of TIRAP-Venus (Fig 2B). Co-expression of untagged MyD88 with TIRAP-Venus had no effect on TIRAP interaction with the receptors. These data are consistent with current knowledge that TIRAP serves as a bridging adaptor for MyD88, and is required for MyD88 interaction with TLRs [8, 9].

Ligand-induced BRET is defined as a change in BRET ratio after addition of a specific ligand, and is used to demonstrate changes in proximity between two proteins as a result of stimulation by the ligand [30]. Cells were transfected with TLR1/2-Rluc8 and either TIRAP-Venus (Fig 2C), or MyD88-Venus and untagged TIRAP constructs (Fig 2D). Pam3CSK4 is a synthetic triacyl lipopeptide and a well-characterized specific ligand for TLR1/2. Addition of Pam3CSK4 to transfected cells was expected to activate the TLR1/2 and induce interaction with TIRAP and MyD88, causing an increase in BRET ratio [5]. However, there was no change in BRET ratio after Pam3CSK4 treatment of cells transfected with either TLR1/2 + TIRAP-Venus (Fig 2C) or TLR1/2 + MyD88-Venus + TIRAP constructs (Fig 2D). Despite very high Pam3CSK4 concentrations being used (10 μg/ml), the BRET ratio was the same as vehicle-only control. Pam3CSK4 was confirmed to be biologically active, as it induced TNF cytokine responses in THP1 cells, a human monocytic cell line. Pam3CSK4 did not induce a TNF response from HEK293FT cells, with or without TLR pathway transfection, likely due to absence of a functional pathway for TNF expression (Fig 2E).

### BRET indicates constitutive proximity between overexpressed proteins

Although no ligand-induced BRET was detected, the high ‘resting’ BRET ratio between TLR1/2-Rluc8 and TIRAP-Venus, and between TLR1/2-Rluc8 and MyD88-Venus in the presence of TIRAP, suggested a constitutive interaction between these proteins. This was unexpected, as it has been generally assumed that TIRAP and MyD88 only interact with TLRs upon ligand binding and receptor activation. To provide further evidence for constitutive TIRAP proximity with TLR1/2, the BRET saturation assay was employed. Increasing concentrations of fluorophore-tagged constructs were transfected, whilst the concentration of luciferase-tagged construct was kept constant [31]. The BRET signal was then plotted as a function of acceptor/donor expression ratio. Specific proximity theoretically results in a saturation curve, compared to a linear or quasi-linear relationship due to random co-localization [31]. These curves can be determined using nonlinear regression model fitting (GraphPad Prism, one site—specific binding). From the saturation BRET curve, a BRET_50_ value can be calculated, which is the fluorescence/luminescence value at 50% of the maximal BRET signal. It has been suggested that a low BRET_50_ is indicative of a direct interaction, whereas a higher BRET_50_ indicates weak or no interaction [32].

**Fig 3:**
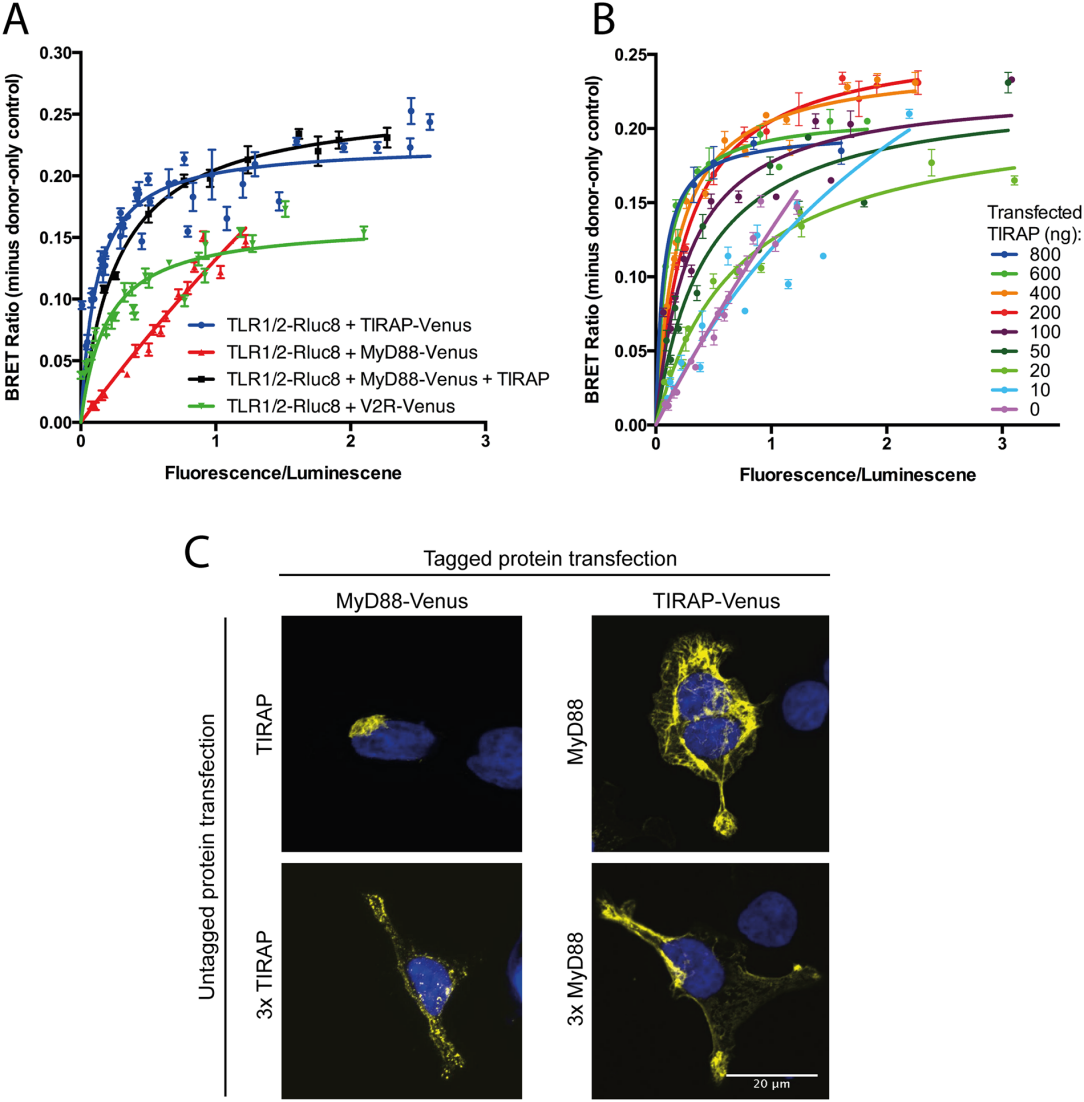
When overexpressed, TIRAP constitutively interacts with TLR1/2 but MyD88 interaction only occurs in the presence of TIRAP. A) HEK293FT cells were transfected with constant (100 ng) TLR1/2-Rluc8 and increasing (0–1000 ng) TIRAP-Venus (blue circles; combined n = 7), MyD88-Venus (red triangles; combined n = 3), MyD88-Venus + TIRAP (black squares, combined n = 2), or V2R-Venus constructs (green inverted triangles, combined n = 3). B) HEK293 cells were transfected with constant (50 ng) TLR1/2-Rluc8 and increasing (0–1000 ng) MyD88-Venus + TIRAP constructs at the concentrations indicated (combined n = 2). Saturation curves were fitted using ‘one site—specific binding’ function on Prism software. Data shown are combined from independent experiments, as indicated. C) HEK293FT cells were transfected with Venus-tagged and untagged MyD88 and TIRAP constructs as indicated, DAPI stained, and imaged by confocal microscopy. Yellow = Venus, blue = nucleus; representative images of two independent experiments.

BRET between TLR1/2-Rluc8 and TIRAP-Venus showed clear saturation, supportive of constitutive proximity between these two proteins (Fig 3A, blue curve). This specificity was further demonstrated by a low BRET_50_ value of 0.117 (Table 1). In contrast, BRET between TLR1/2-Rluc8 and MyD88-Venus had a linear relationship indicating a clear lack of proximity (Fig 3A, red line). This was consistent with confocal microscopy experiments (Fig 1F), which demonstrated that in the absence of TIRAP, overexpressed MyD88 was localized intracellularly away from the plasma membrane. In contrast, when untagged TIRAP was co-expressed with MyD88-Venus, clear saturation was observed, demonstrating again that MyD88 requires TIRAP to interact with TLR1/2 (Fig 3A, black curve). V2R-Venus was used as a negative control, as this receptor was not expected to specifically interact with TLR1/2. Intriguingly, a saturation curve for TLR1/2-Rluc8 and V2R-Venus was observed, although this was lower than that of TLR1/2 and TIRAP (Fig 3A, green curve). The BRET_50_ value was also higher for TLR1/2-Rluc8 and V2R-Venus (0.193), which are not known to have any meaningful biological interactions. This effect with saturation BRET can occur with overexpression of proteins that might co-localize at the cell membrane without any functional consequences [33], and again illustrates the need for careful interpretation of saturation BRET data. This being said, the clear linear relationship for TLR1/2-Rluc8 + MyD88-Venus with the switch to a clear saturation curve upon co-expression of TIRAP illustrates the utility of this approach under certain circumstances.

**Table 1:** BRET_50_ values calculated from BRET saturation curves show MyD88 and TLR2 interactions are dependent on TIRAP concentration.

	*BRET*_*50*_	*BRET*_*50*_ *SEM*	*BRET*_*max*_	*BRET*_*max*_ *SEM*
**TLR-Rluc8 +** **TIRAP-Venus**	0.117	0.011	0.23	0.004
**TLR-Rluc8 +** **V2R-Venus**	0.193	0.022	0.16	0.005
**TLR-Rluc8 + MyD88-Venus + TIRAP**				
*TIRAP (ng DNA)*				
0	ND*	ND*	ND*	ND*
10	ND*	ND*	ND*	ND*
20	0.71	0.063	0.21	0.0076
50	0.43	0.081	0.23	0.014
100	0.29	0.038	0.23	0.0087
200	0.27	0.018	0.26	0.0041
400	0.19	0.014	0.24	0.0041
600	0.090	0.0097	0.21	0.0044
800	0.066	0.022	0.20	0.0070

SEM—standard error

BRET_max_—maximum BRET ratio, calculated from the fitted curve

BRET_50_—fluorescence/luminescence value at 50% BRET_max_, calculated as the equilibrium binding constant (Kd) from the fitted curve

* Curves for 0 and 10 ng TIRAP DNA constructs are sufficiently linear that curves cannot be fitted with enough accuracy to determine appropriate BRET_max_ and BRET_50_ values.

To further investigate the role of TIRAP in bridging MyD88 and TLR1/2, BRET saturation assays were performed as before, with constant TLR1/2-Rluc8 and TIRAP constructs co-transfected with increasing MyD88-Venus construct. Multiple individual curves were generated, each with different amounts of constant TIRAP DNA construct transfection. As TIRAP construct concentrations increased, the linear relationship changed to a saturation curve, indicating that a minimum expression level of TIRAP was required to allow constitutive interaction of MyD88 with TLR1/2 (Fig 3B). Weak interactions between TLR1/2-Rluc8 and MyD88-Venus, in the absence of TIRAP, were highlighted by an incalculable BRET_50_ value (Table 1). However, when TIRAP was co-transfected with MyD88-Venus in increasing DNA construct concentrations, the calculable BRET_50_ value decreased (Table 1). This indicated specific proximity when more than 10 ng of TIRAP construct was transfected. The saturation BRET results suggest that a threshold amount of TIRAP is needed to allow MyD88 to interact with TLR1/2. Indeed, when MyD88-Venus construct was co-transfected with untagged TIRAP construct at equal concentrations, there was no effect on MyD88 localization seen by confocal microscopy, but when TIRAP construct was transfected at three-fold higher concentration, MyD88 localization changed to small punctate staining at or near the plasma membrane (Fig 3C). In contrast, co-transfection of MyD88 construct had no effect on TIRAP localization (Fig 3C). Thus, confocal microscopy confirms that a threshold amount of TIRAP is necessary to redistribute MyD88.

### NanoBRET allows investigation with lower protein expression, but constitutive proximity is still observed

**Fig 4:**
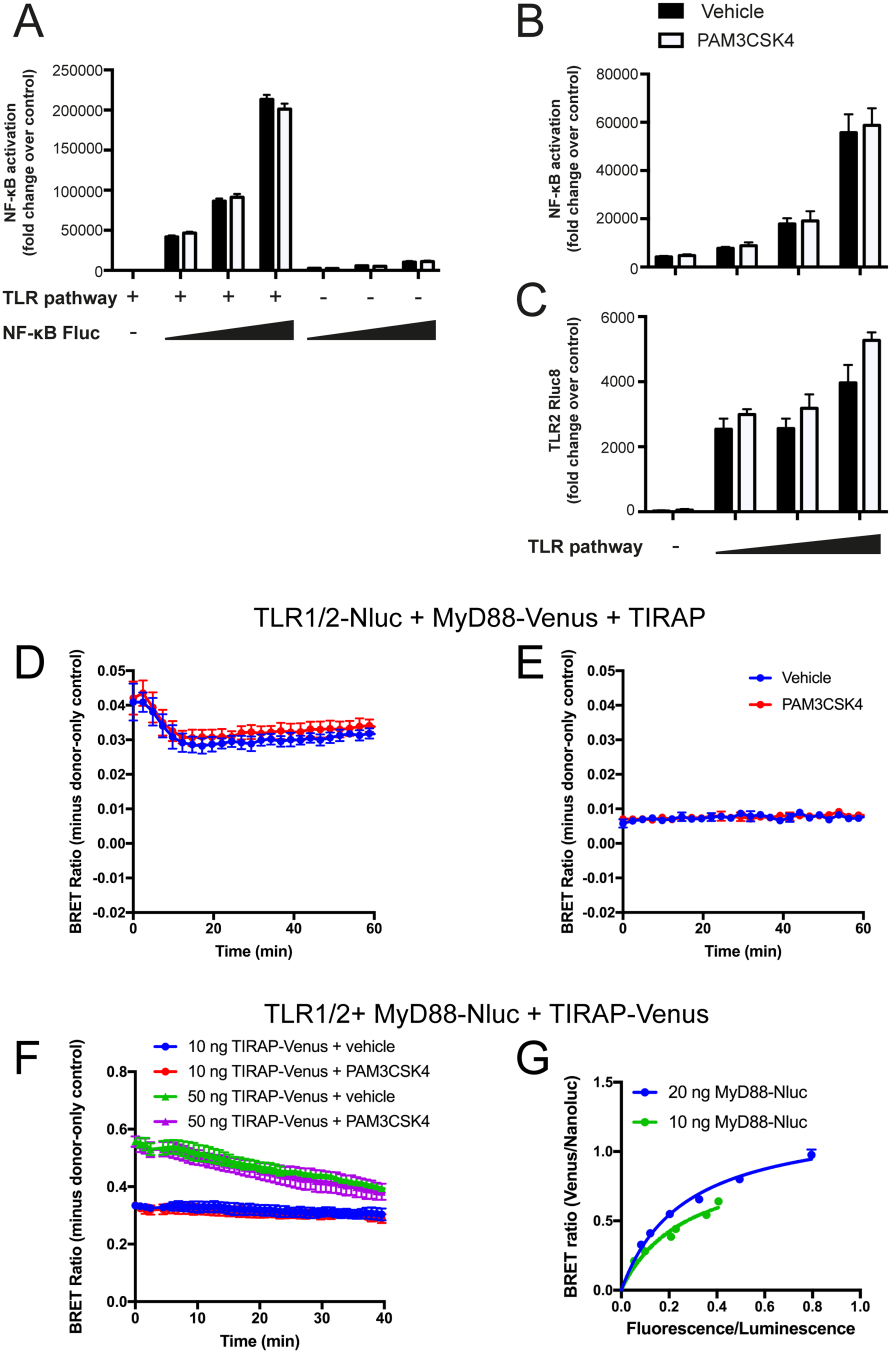
Modified BRET using Nluc allowed reduction in protein expression levels, but did not enable observation of ligand-induced BRET ratio. A) HEK293FT cells were transfected with 100 ng of TLR1/2, MyD88 and TIRAP constructs (TLR pathway) and 0, 100, 200 or 400 ng of NF-κB luciferase constructs, and treated with 1 μg/ml Pam3CSK4. NF-κB luciferase levels measured after 24 hr; representative experiment of n = 3. B and C) HEK293FT cells were transfected with 200 ng of NF-κB-Fluc constructs and 0, 25, 50 or 100 ng of TLR1/2, MyD88 and TIRAP constructs (TLR pathway), and treated with 1 μg/ml Pam3CSK4. NF-κB reporter Fluc luciferase (B) and TLR-Rluc8 luciferase (C) levels measured after 24 hr; representative experiment of n = 2. HEK293FT cells transfected with D) 50 ng TLR1 + 50 ng TLR2-Nluc + 50 ng MyD88-Venus + 100 ng TIRAP constructs, or E) 5 ng TLR1 + 5 ng TLR2-Nluc + 5 ng MyD88-Venus + 10 ng TIRAP constructs. Cells were stimulated with 1 μg/ml Pam3CSK4 (red) or vehicle control (blue) and BRET measured over time. Data shown are donor-only-subtracted BRET ratio; graphs are representative of three independent experiments. F) HEK293FT cells were transfected with 100 ng TLR1 + 100 ng TLR2 + 10 ng MyD88-Nluc and 10 ng (circle) or 50 ng (triangle) of TIRAP-Venus constructs. Cells were stimulated with 1 μg/ml Pam3CSK4 (red and purple) or vehicle control (blue and green) and BRET measured over time. Data shown are donor-only-subtracted BRET ratio; graphs are representative of two independent experiments. G) HEK293FT cells were transfected with constant (100 ng) TLR1/2 and MyD88-Nluc constructs (20 ng, blue; or 10 ng, green), and increasing (0–600 ng) TIRAP-Venus construct. Saturation curves were fitted using ‘one site—specific binding’ function on Prism software.

The constitutive proximity between TLR1/2, TIRAP and MyD88 seen in the BRET experiments in the absence of ligand was unexpected, as previous work indicated that MyD88 only interacts with TIRAP upon ligand stimulation [34]. However, it was also shown that overexpression of MyD88 induced constitutive NF-κB activation [35] and ligand-independent interaction between TLR4 and MyD88 [36]. Indeed, co-transfection of the TLR pathway proteins with a firefly luciferase (Fluc) NF-κB reporter construct showed an NF-κB response that was dose dependent on the TLR pathway components, but was independent of Pam3CSK4 (Fig 4A–4C). Therefore, the constitutive interaction between TLR1/2, TIRAP and MyD88 observed with BRET might be due to overexpression of these proteins, resulting in spontaneous interaction/oligomerization.

Overexpression of tagged constructs tends to be necessary when performing BRET with Rluc8 and Venus, because bright luminescent signals are required for detection. However, Nanoluc (Nluc), a new luciferase enzyme that is significantly brighter than Rluc8, was recently applied to BRET studies [37]. Use of Nluc can lower the limit of detection for BRET, essentially allowing sensitive BRET measurement with reduced expression levels of the enzyme (termed NanoBRET). In an attempt to overcome the potentially non-specific constitutive interactions between TLR1/2, MyD88 and TIRAP due to protein overexpression, the BRET assay was repeated using Nluc in place of Rluc8.

NanoBRET using TLR1/2-Nluc and MyD88-Venus or TIRAP-Venus allowed a 10-fold reduction in construct expression whilst still providing detectable BRET signal. Cells transfected with 5 ng of TLR2-Nluc construct (Fig 4E) allowed for a stably detectable BRET signal compared to cells transfected with 50 ng of TLR2-Nluc construct (Fig 4D). Due to the brighter light emission of Nluc compared to Rluc8 [37], lower DNA construct concentrations were used for transfection in order to prevent signal saturation. However, ligand-induced BRET remained undetectable, since treatment with Pam3CSK4 did not induce a change in BRET ratio compared to vehicle only control (Fig 4D and 4E). Therefore, simply exchanging Rluc8 for Nluc in this experimental setup was not sufficient to improve measurement of ligand-induced receptor-adaptor protein interactions.

**Fig 5:**
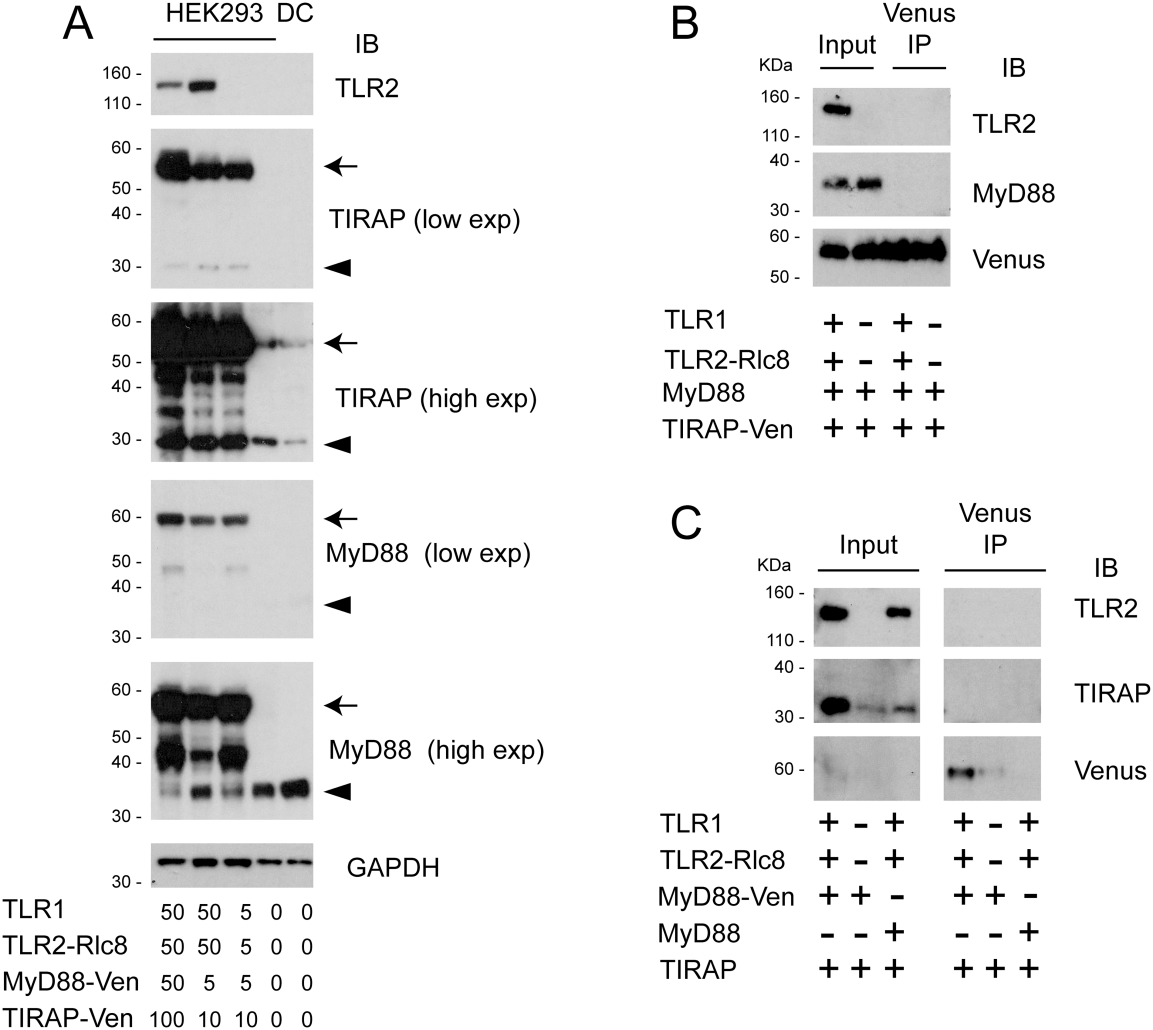
Immunoprecipitation does not show constitutive interactions between overexpressed TLR1/2, TIRAP and MyD88. A) HEK293FT cells were transfected as indicated, and cell lysates were analysed by Western blotting. High and low exposure images show both Venus-tagged (arrows) and endogenous (triangles) MyD88 and Venus in the same blot. B and C) HEK293FT cells were transfected with 100 ng of DNA constructs as indicated. Immunoprecipitation of Venus-tagged protein was performed, and analysed by Western blot, along with input samples.

Since the current understanding is that TIRAP and MyD88 should only interact upon ligand activation of a receptor, the monitoring of interactions between these two proteins was attempted as a strategy for assessing ligand-induced BRET. MyD88, which based on previous experiments seemed to be most prone to oligomerization, was tagged with Nluc to allow lowest possible expression levels. Cells were co-transfected with MyD88-Nluc and TIRAP-Venus construct along with untagged TLR1/2 construct, and the BRET ratio was measured in response to Pam3CSK4 ligand or vehicle only control. Stimulation of TLR1/2 with Pam3CSK4 was expected to induce interaction between MyD88-Nluc and TIRAP-Venus, however, no change in BRET was observed (Fig 4F). Despite the concentration of transfected DNA construct being reduced ten-fold, constitutive BRET was still apparent between MyD88-Nluc and TIRAP-Venus, indicated by a saturation BRET curve (Fig 4G). Therefore, it appears that constitutive proximity between MyD88 and TIRAP still occurred when these proteins were overexpressed in cells, even if low transfection concentrations of DNA construct were used. In fact, transfection of TIRAP-Venus and MyD88-Venus constructs, even at the low concentrations used in NanoBRET, still produced overexpression of these proteins in HEK293FT cells (Fig 5A). These remained significantly higher than the endogenous protein levels in human primary dendritic cells.

### Co-immunoprecipitation studies did not confirm constitutive interaction between TLR1/2 and adaptors

In order to further investigate the constitutive interaction with TLR1/2 and TIRAP/MyD88 observed by BRET, co-immunoprecipitation (IP) was employed. Venus IP was performed on cells expressing Venus-tagged TIRAP and untagged MyD88 (Fig 5B), or Venus-tagged MyD88 and untagged TIRAP (Fig 5C), with and without TLR1/2-Rluc8. Cells transfected with TLR1/2-Rluc8 and untagged MyD88 and TIRAP constructs were used as a negative control (Fig 5C). In contrast to the BRET data, the IP experiments did not demonstrate constitutive interactions between the TLR pathway proteins.

## Discussion

The downstream signalling from TLRs is hinged on interactions between receptors and adaptor proteins via their TIR domains [4]. Currently, there are no suitable methods available to specifically monitor these interactions. BRET is a sensitive method for detection of specific protein-protein interactions in live cells, and was applied to investigate the binding between TLRs and adaptors. To our knowledge, BRET has never before been used to study TLRs, with the closest example being the use of FRET to analyse the dimerization of TLR4 [38]. However, BRET requires the overexpression of the proteins of interest, which can be a confounding factor if the protein activity is sensitive to expression stoichiometry. Furthermore, addition of luciferase and fluorophore tags can, in some cases, alter the structure of the target proteins and subsequently affect their localization or function. Therefore, the expression and subcellular localization of tagged proteins must always be evaluated prior to application in BRET experiments.

The BRET and confocal microscopy analysis in this study demonstrated that the TLR2-Rluc8 and TIRAP-Venus were expressed and correctly localized at the plasma membrane. However, MyD88-Venus showed intracellular localization, which has been reported for artificially overexpressed MyD88 [9, 26, 28]. Previous work has claimed that the unique subcellular localization of transfected MyD88 constitutes the natural distribution of the protein [28]. Yet, more recently it has been shown that endogenous MyD88 in macrophages is found in small speckle-like formations throughout the cytoplasm, and condense into larger structures upon TLR stimulation with ligands [39]. Thus, the observed distribution of MyD88 when overexpressed in cells may be an artefact occurring due to the death domains of MyD88 being prone to oligomerization. Co-expression of TIRAP and MyD88-Venus changed the subcellular localization of MyD88-Venus to smaller speckle-like formations at the plasma membrane, indicating that MyD88-Venus was functionally interacting with TIRAP. The BRET experiments conducted in this study showed that, similar to the bridging role of TIRAP in TLR4-MyD88 signalling [8, 9, 40], TIRAP is also required for MyD88 interaction with TLR2. Interestingly, the requirement for three-fold higher transfection amount of TIRAP to achieve this redistribution of MyD88 indicated that there was a minimum threshold of TIRAP required to translocate MyD88, likely by disruption of MyD88 oligomerization to favour TIRAP:MyD88 complex formation. This was supported by saturation BRET studies, which showed that the interaction between TLR1/2 and MyD88 was dependent on TIRAP expression levels. This constitutes a novel concept that TIRAP expression levels in cells could be a rate-limiting step in MyD88-dependent signalling downstream of TLR2, and potentially of TLR4.

Ligand-induced BRET is important to show that the interactions seen between tagged proteins are functional and biologically valid [23]. Despite several strategies used in this study for tagging the proteins of interest, ligand-induced BRET was not observed when cells were treated with the TLR1/2 ligand Pam3CSK4 [3]. Saturation BRET has been extensively used to investigate oligomerization between GPCRs [31, 33], and our experiments demonstrated that the proteins analysed were constitutively proximal, even at low transfection concentrations. This was despite the use of Nluc to increase BRET sensitivity and reduce protein expression levels [37]. Indeed, Western blot analysis showed that protein levels of ectopically expressed TIRAP and MyD88 remained significantly higher than endogenous proteins found in immune cells (Fig 5A). Therefore, because the tagged proteins demonstrated constitutive proximity in the absence of ligand, measurement of ligand-induced BRET was not achievable.

Most previous studies on interactions between TLRs, TIRAP and MyD88 have used protein overexpression systems, which induced ligand-independent activation of transcription factors such as NF-κB [6, 11, 19, 41]. Additionally, it has been reported that overexpression of TLR4 and TIRAP caused ligand-independent interactions [6]. Only recently has there been a study demonstrating the ligand-induced interaction of endogenous MyD88 and TIRAP [34]. The study by Bonham et al. clearly showed that MyD88 and TIRAP interacted only after stimulation with TLR ligands LPS and CpG, although the authors did not investigate native interactions between TLRs and TIRAP. Therefore, it is more likely that the ligand-independent interactions between MyD88, TIRAP and TLR1/2 observed with BRET are artefacts due to the overexpression of these proteins. This is further supported by the IP studies, which did not show constitutive interactions between the proteins of interest. However, it is important to note that IP studies are not without limitations, as they usually only capture strong protein-protein interactions, whereas transient or low affinity interactions will likely not be captured due to stringent assay conditions. Therefore, some biologically ‘real’ protein-protein interactions can be lost during membrane disruption and/or under IP conditions, and thus cannot be demonstrated with this method.

Ligand induced interactions between endogenous TLR1/2 and TIRAP have not yet been investigated. It is possible that TLR1/2-TIRAP protein complexes could occur naturally in cells in the absence of ligand-induction, with ligand binding causing conformational changes in the complex that initiate the downstream signalling cascade. Another possibility is that the proteins are located within close proximity to each other at resting state, and ligand binding rapidly induces interactions to initiate signalling. The actuality of either of these scenarios remains to be determined, although it will likely not be achieved using standard BRET techniques. Instead, to conclusively determine the native resting interactions between these proteins, studies need to be undertaken with endogenous proteins for immunoprecipitation and/or immunofluorescence studies. Alternatively, *in situ* knock-ins of tags using CRISPR/Cas9 gene editing could be used to insert the luciferase and fluorophore genes, allowing the study of TLR-MyD88-TIRAP complexes at physiological expression levels [42]. This technique has been used recently for BRET, where knock-in of Nluc overcame the need for overexpression of donor fusion proteins [43]. However, the genomic insertion of large genes like that of the Venus fluorophore remain a challenge, even with CRISPR/Cas9 gene editing.

In summary, our data confirm that TIRAP is essential for MyD88 interaction with TLR1/2, constituting a rate-limiting component of TLR1/2-MyD88 interaction. Furthermore, constitutive proximity/interaction between TLR1/2 and adaptors was observed with BRET, although this is likely to be an artefact of protein overexpression. These findings highlight the need for caution when studying the TLR pathway through ectopic protein expression. Despite the challenges with experiments using endogenous protein expression, these are likely to be critical to our future understanding of genuine protein-protein interactions in cells.

## Materials and methods

### Constructs

Human TLR1, TLR2, and TIRAP genes were purchased from Invivogen, and all cloning was performed by GeneArt (Life Technologies). The MyD88 gene was synthesised *de novo* due to high CG content. Genes were sub-cloned into pcDNA3.1 vectors containing C-terminal Rluc8, Nluc, or Venus tags. The Kras-Venus construct was kindly provided by Nevin Lambert. Constructs were purified from TOP10 *E*. *coli* (Life Technologies) using an endotoxin-free Maxiprep kit (Qiagen).

### Cell culture and transfection

HEK293FT cells (Thermo Fisher Scientific) were maintained in DMEM (Dulbecco’s modified Eagle’s medium, 4.5 g/L D-glucose, 40 mM sodium bicarbonate, 100 U/ml penicillin and 100 μg/ml streptomycin) supplemented with 10% heat-inactivated FCS, at 37°C in a 5% CO_2_ humidified incubator. Cultures were maintained at sub-confluency and passaged every two to three days using 2.5% trypsin treatment (Gibco).

HEK293FT cells were transfected using GeneJuice (Novagen) according to manufacturer’s instructions. Briefly, 6 x 10^5^ cells/well were added to 6-well plates 24 hr prior to transfection. Transfection was done when cells were approximately 50% confluent, by mixing up to 1.2 μg of expression plasmid with 100 μl of serum-free medium and 4 μl of GeneJuice. After 10 min incubation at RT, the GeneJuice/plasmid mixture was added to cells dropwise. Cells were incubated for 24–48 hr prior to assaying. The concentration of transfected DNA was always equalised between samples using pcDNA3.1 empty vector.

### Confocal microscopy

HEK293FT cells were transfected as described. 24 hr post-transfection, cells were detached using trypsin treatment and 8 x 10^5^ cells added to 6-well plates containing poly-L-lysine-coated glass coverslips. After 24 hr, wells were washed twice with PBS prior to cell fixation using 4% paraformaldehyde in fixative buffer (10 mM KCl, 274 mM NaCl, 8 mM NaHCO_3_, 0.8 mM KH_2_PO_4_, 4 mM MgCl_2_, 10 mM PIPES pH 7.2, 4 mM EGTA, 11 mM glucose) for 5 min at 37°C. Cells were permeabilised with 0.15% Triton-X 100 in fixative buffer for 20 min and washed once with 0.1 M glycine and four times with TBS for 10 min each. Coverslips were blocked with 1% BSA in TBS for 20 min. Coverslips were washed twice with 1% BSA in TBS for 10 min, twice with TBS for 5 min, and mounted onto glass slides using Prolong Gold with DAPI (Thermo Fisher). Cells were imaged on a Zeiss LS 780 confocal microscope.

### Bioluminescence resonance energy transfer assays

HEK293FT cells were transfected as described, and 24 hr post-transfection cells were detached using trypsin treatment and washed once with PBS. Cells were re-suspended at 1 x 10^6^ cells/ml in phenol red-free DMEM supplemented with 5% FCS and 25mM HEPES. 1 x 10^5^ cells/well were added to poly-L-lysine-coated opaque white 96-well plates and incubated for a further 24 hr. Medium was exchanged with medium containing 5 μM coelenterazine *h* and assays carried out immediately. In some instances, 10 μl Pam3CSK4 (Pam3CysSerLys4, synthetic triacylated lipopeptide; Invivogen) in medium was added to wells a few minutes into the assay measurement, to indicated final concentrations. For NanoBRET experiments using Nluc constructs, samples were prepared as above, but furimazine (Promega) diluted 1:500 in medium was added to cells immediately prior to assay.

BRET measurements at 37°C utilised the PHERAstar plate reader and software (BMG Labtech). Emissions were simultaneously measured at 400–475 nm for Rluc8 or Nluc (donor) and at 520–540 nm for Venus (acceptor). BRET was calculated by subtracting the ratio of emission through the acceptor wavelength window over emission through the donor wavelength window. In some instances, data were presented as BRET ratio minus donor-only control, where donor-only control was cells transfected with luciferase-tagged DNA constructs only, to control for background luminescence.

### BRET saturation assay

The BRET saturation assay was performed as described, with varying concentrations of transfected DNA constructs, as indicated in figures. For direct fluorescence measurements, transfected cells used in the BRET assay were also plated at 0.5 x 10^5^ cells/well in black 96-well plates. Fluorescence after light excitation was measured on an EnVision 2102 plate reader (PerkinElmer Life Sciences) using a 485/14 excitation filter, 535/25 emission filter, and D505 mirror. Luminescence data points were selected at time points when maximal luminescence was obtained. BRET ratios at that same time point were used. Data were plotted as BRET ratio vs. fluorescence/luminescence, and curves were fitted using ‘one site—specific binding’ function on Prism software (GraphPad). BRET_50_, corresponding to the fluorescence/luminescence value at 50% of the maximum BRET ratio, was calculated as the equilibrium dissociation constant (Kd) from the fitted curve. In order to determine if the relationship was linear or saturated, all curves were compared to a ‘line through origin’ curve (null hypothesis indicating a linear relationship), and the null hypothesis was rejected if the p-value was less than 0.05.

### Western blotting and immunoprecipitation

HEK293FT cells transfected as for BRET assay, THP1 cells, or human primary monocyte-derived dendritic cells were lysed as described previously [44]. Protein concentrations were determined using the Pierce BCA Protein Assay Kit (Thermo Fisher) and equalized between samples. For Venus immunoprecipitation, Venus cross-reacting GFP-Trap beads (Chromotek) were used according to manufacturer’s instructions. Samples and Novex Sharp protein size marker (Thermo Fisher) were separated by SDS-PAGE using the NuPAGE system with 4–12% Bis-Tris gels (Thermo Fisher) and transferred onto nitrocellulose membranes using the Criterion transfer system (Bio-Rad). Membranes were probed with antibodies against TLR1 (Cell Signalling Technology), TLR2 (D7G9Z, Cell Signalling Technology), MyD88 (D80F5, Cell Signalling Technology), TIRAP (EPR3509, Abcam), GFP (cross-reacts with Venus, 13.1/7.1, Roche), or Renilla luciferase (EPR17791, Abcam), followed by secondary probing with HRP-conjugated anti-mouse or anti-rabbit antibodies (GE Healthcare) as previously described [44]. HRP-conjugated antibodies against β-actin (AC-15, Sigma) and GAPDH (Proteintech) were used to demonstrate even lane loading.

### TNF ELISA

HEK293FT cells transfected as for BRET assay, or THP1 cells were plated in 96-well plates as described above. After 24 hr, cells were treated with vehicle or Pam3CSK4 as indicated and incubated for a further 24 hr. Subsequently, cell culture supernatant was collected and TNF ELISA (Invitrogen eBioscience Human TNF alpha ELISA Ready-SET-Go! Kit) performed according to manufacturer’s instructions.

### NF-κB luciferase

HEK293FT cells were transfected as for BRET assays, and additionally with NF-κB Firefly luciferase reporter DNA construct as indicated. After 48 hr, cells were lysed using Passive Lysis Buffer (Promega), and Renilla and Firefly luciferase activity were measured sequentially using the Dual-Glo Luciferase Assay System (Promega) on a GloMax luminometer (Promega), according to manufacturer’s instructions.
